# The Evolving Role of Zika Virus Envelope Protein in Viral Entry and Pathogenesis

**DOI:** 10.3390/v17060817

**Published:** 2025-06-06

**Authors:** Ashkan Roozitalab, Jiantao Zhang, Chenyu Zhang, Qiyi Tang, Richard Y. Zhao

**Affiliations:** 1 Department of Pathology, University of Maryland School of Medicine, Baltimore, MD 21201, USA; aroozitalab@som.umaryland.edu (A.R.); chenyu.zhang@som.umaryland.edu (C.Z.); 2 Department of Microbiology, Howard University College of Medicine, Washington, DC 20059, USA; qiyi.tang@Howard.edu; 3 Department of Microbiology and Immunology, University of Maryland School of Medicine, Baltimore, MD 21201, USA; 4 Institute of Global Health, University of Maryland School of Medicine, Baltimore, MD 21201, USA; 5 Institute of Human Virology, University of Maryland School of Medicine, Baltimore, MD 21201, USA; 6 Research and Development Service, VA Maryland Heath Care System, Baltimore, MD 21201, USA

**Keywords:** Zika virus, envelope protein, viral entry, microcephaly, inhibitors, neutralizing antibodies, antibody-dependent enhancement, ancestral and epidemic viral strains, African and Asian lineages

## Abstract

Zika virus (ZIKV) was first discovered in Uganda’s Zika Forest in 1947. The early African viruses posed little or no health risk to humans. Since then, ZIKV has undergone extensive genetic evolution and adapted to humans, and it now causes a range of human diseases, including neurologically related diseases in adults and congenital malformations such as microcephaly in newborns. This raises a critical question as to why ZIKV has become pathogenic to humans, and what virological changes have taken place and enabled it to cause these diseases? This review aims to address these questions. Specifically, we focus on the ZIKV envelope (E) protein, which is essential for initiating infection and plays a crucial role in viral entry. We compare various virologic attributes of E protein between the ancestral African strains, which presumably did not cause human diseases, with epidemic strains responsible for current human pathogenesis. First, we review the role of the ZIKV E protein in viral entry and endocytosis during the viral life cycle. We will then examine how the E protein interacts with host immune responses and evades host antiviral responses. Additionally, we will analyze key differences in the sequence, structure, and post-translational modifications between African and Asian lineages, and discuss their potential impacts on viral infection and pathogenesis. Finally, we will evaluate neutralizing antibodies, small molecule inhibitors, and natural compounds that target the E protein. This will provide insights into the development of potential vaccines and antiviral therapies to prevent or treat ZIKV infections and associated diseases.

## 1. Introduction

### 1.1. Zika Virus (ZIKV): A Brief History

The Zika virus (ZIKV) is an enveloped, positive-sense, single-stranded RNA (+ssRNA) virus belonging to the *orthoflavivirus* genus within the family of Flaviviridae, which includes other medically significant arboviruses such as West Nile virus (WNV), Dengue virus (DENV), Japanese Encephalitis virus (JEV), and Chikungunya virus (CHIKV) [1]. These viruses are transmitted to humans and other animals through the bite of infected arthropods, such as mosquitoes *Aedes aegyptis*; thus, they are referred to as “arboviruses”, short for “arthropod-borne viruses”. Arboviruses cause a range of illnesses, from mild to severe, with serious health consequences. For example, WNV is prevalent worldwide and is the most common arbovirus in the U.S., potentially leading to severe neurological diseases such as encephalitis and meningitis, although many infections remain asymptomatic.

ZIKV was first isolated from a rhesus monkey in Uganda’s Zika Forest in 1947 [2]. Early studies showed that infected monkeys displayed mild or no symptoms, but mice younger than two weeks were highly susceptible to intraperitoneal (i.p.) inoculation; whereas older mice rarely became infected, likely due to their fully developed blood-brain barriers (BBB), which prevented ZIKV from accessing the brain. Subsequent research confirmed ZIKV’s ability to infect the central nervous system (CNS) in mice [3].

Although ZIKV is primarily transmitted by *Aedes* mosquitoes to both humans and animals, it initially appeared less harmful to humans [4]. The first human ZIKV infection was recorded in 1952 [5], and since then, ZIKV antibodies have been detected in human sera from various countries [6], although no severe diseases were initially linked to these infections. ZIKV can also infect humans via non-vector-borne routes, including sexual transmission, maternal-fetal transmission, and blood transfusions [7,8,9]. Most individuals infected with ZIKV are asymptomatic or experience mild symptoms such as rash, fever, joint pain, and conjunctivitis.

The first laboratory strain of ZIKV, MR766 (ZIKV_MR766_), was named after the febrile rhesus monkey number 766 from which it was first isolated in 1947 in the Zika Forest of Uganda, during studies of yellow fever [5,10]. This strain, now referred to as the ancestral or historical ZIKV strain, has been widely used for research into the virus’s biology, pathogenesis, and vaccine development. ZIKV_MR766_ demonstrated high neurotropism in mice, with the virus being recoverable only from infected mouse brains [4].

Since its initial discovery, ZIKV has spread globally beyond Africa, with cases reported in Asia and the Americas [1,6]. Over the past seventy plus years, ZIKV has migrated from Africa to Asia, across the Pacific, and eventually to the Americas. Early ZIKV infections, primarily in Africa and Asia, were not linked to severe disease, and its spread eastward from Africa included countries such as Pakistan, Malaysia, and Indonesia [11,12,13]. Outbreaks occurred in these regions during 1977–1978, but they were relatively small. However, as the virus crossed the Pacific Ocean, significant outbreaks occurred on Pacific islands, including Yap Island in Micronesia in 2007 and in French Polynesia in 2013–2014. These outbreaks were noteworthy due to the increased incidence of Guillain–Barré syndrome-like symptoms and, in some cases, birth defects [9,14]. The virus reached the Americas in 2015, with a major outbreak starting in Brazil. From there, ZIKV spread rapidly across Central and South America, and the Caribbean. In 2016, the World Health Organization (WHO) declared ZIKV a global public health emergency [15]. During this outbreak, ZIKV infections were reported in eighty-five countries, territories, or subnational areas, affecting an estimated 1.5 million individuals globally, according to the WHO. Brazil had the hardest hit, with estimates ranging from 440,000 to 1.3 million cases by December 2015 [16]. Although the spread of ZIKV has declined since 2017, the virus remains a global threat, with the potential for significant outbreaks again. A recent example is the 2021 outbreak in Kerala, India, during the second wave of the COVID-19 pandemic [17]. As of May 2024, ZIKV transmission had been reported in ninety-two countries and territories, according to the WHO. The history of ZIKV epidemiology and evolution clearly demonstrates that the virus has gradually adapted to humans as a host. Over nearly eighty years, changes within the viral genome have driven the emergence of more pathogenic ZIKV strains, contributing to its increasing threat to human health [1,18].

### 1.2. The Structure and Genomic Organization of ZIKV

**Figure 1:**
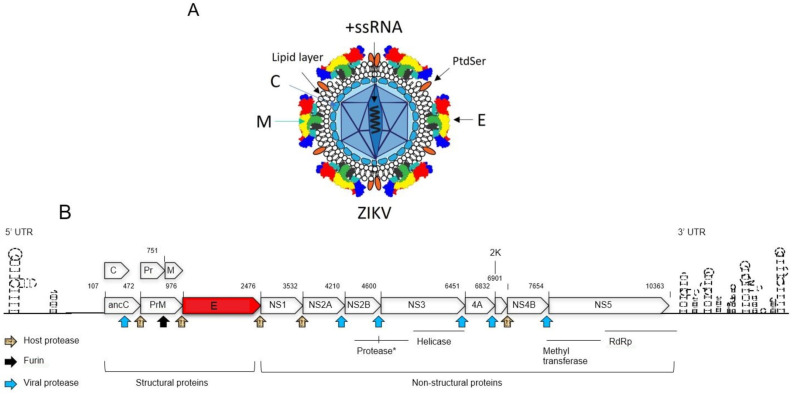
Schematic representation of the ZIKV viral particle and genome. (**A**) The Zika viral particle is surrounded by a lipid envelope. The E protein, organized into “rafts” of three E protein dimers lying parallel to each other, covers most of the virion surface. Beneath the E protein lies the M protein. The C protein forms the shell that encloses the viral +ssRNA genome. Phosphatidylserine (PtdSer), a phospholipid, is interlocked with E proteins to form “raft-like” structures on the viral surface. (**B**) This diagram illustrates the genomic organization of ZIKV, with each viral protein depicted according to its relative position within the RNA genome. Protease cleavage sites are indicated by arrows, representing cleavage by the viral protease, host protease, and furin protease. The numbers above each protein product denote their start and end positions within the genome. The abbreviations used are as follows: anaC (anchored capsid protein C), C (capsid protein C), prM (precursor membrane protein), M (membrane protein), Pr (protein pr), E (envelope protein), NS (nonstructural protein), 2K (signal peptide 2K), and UTR (untranslated region). The viral protease consists of the N-terminal domain of NS3 and the C-terminal domain of NS2B, as described in the text (*). The C-terminal region of NS3 encodes a helicase, while NS5 contains a methyltransferase (MTase) at its N-terminal end and an RNA-dependent RNA polymerase (RdRp) at its C-terminal end. This diagram is adapted from [1].

The structure and morphology of ZIKV closely resemble those of other orthoflaviviruses, such as DENV [19]. Specifically, the viral surface proteins are arranged in an icosahedral-like symmetry. ZIKV particles measure approximately 40–65 nm in diameter, with surface projections ranging from 5–10 nm [19], resulting in an average overall size of 45–75 nm. As an enveloped virus, ZIKV is surrounded by a lipid envelope derived from the host cell membrane. Inside this envelope, the viral RNA genome is encased within a protein capsid, approximately 25–30 nm in diameter. This capsid is surrounded by a lipid bilayer primarily composed of three structural proteins: the envelope (E) protein, the membrane (M) protein, and the capsid (C) protein. The E protein covers most of the virion surface, organized into “rafts” of three E protein dimers lying parallel to each other. Beneath the E protein lies the M protein, which plays a crucial role in viral maturation. The C protein forms the shell that encloses the viral RNA genome (Figure 1A).

ZIKV’s genome is +ssRNA of approximately 10.79 kb in size (Figure 1B) [1,19]. It contains a single open reading frame (ORF) flanked by two non-coding regions (NCRs): the 5′ NCR (107 nucleotides) and the 3′ NCR (428 nucleotides) [1,20]. The ZIKV genome encodes a polyprotein that undergoes post-translational processing to produce three structural proteins (C, prM, and E) and seven non-structural proteins (NS1, NS2A, NS2B, NS3, NS4A, NS4B, and NS5), which are necessary for viral replication and assembly [19,21,22]. The non-structural proteins are processed to yield four viral enzymes: proteases (PR), helicase, methyltransferase (MTase), and RNA-dependent RNA polymerase (RdRP). These enzymes play essential roles in viral genome replication, assembly, and evasion of host immune defenses [19]. The structural proteins (C, prM, and E) are essential for the formation of the viral capsid and envelope. The ZIKV C protein plays a crucial role in packaging the viral RNA genome and assembling new virions [23]. In addition, The ZIKV C protein also functions as a viral suppressor of RNA interference by directly interacting with and inhibiting the endoribonuclease activity of host Dicer enzymes in human neural stem cells (NSCs) [24]. The mature C protein is generated when the viral PR cleaves the anchor-C (anaC) protein. This cleavage also triggers the splitting of the precursor membrane (prM) protein, resulting in the mature M protein, and a Pr protein [25,26,27]. Furin cleavage of prM into M in the trans-Golgi network (TGN) before release of mature virions leads to the formation of mature, infectious viral particles [28,29]. The M protein stabilizes the E protein by locking it into a fixed mature conformation, thereby preventing premature membrane fusion during transit through the secretory pathway [30]. Research findings demonstrated that a single amino acid (a.a.) change (S139N) in the ZIKV prM protein, found in most epidemic strains, significantly increases neurovirulence in neonatal mice and induces greater cell death in human neural progenitor cells (hNPCs) compared to the N139S mutant or pre-epidemic strains [23,24]. The E protein plays a key role in binding to host cell receptors and mediating fusion with the host cell membrane during viral entry and endocytosis [31]. ZIKV non-structural proteins carry out essential functions in viral replication, immune evasion, and pathogenesis. NS1 is crucial for viral replication and helps the virus evade the host immune system [32]. The NS2B-NS3 protease is essential for ZIKV replication and maturation, as NS2B acts as a cofactor enabling NS3 to cleave the viral polyprotein into functional non-structural proteins [33]. Functional studies have demonstrated that NS4A and NS4B modulate host immune responses and are associated with neurodevelopmental damage [34]. NS5, the largest ZIKV protein, contains both RdRp and MTase domains, essential for viral RNA synthesis and capping [32].

### 1.3. The Viral Infection Cycle

**Figure 2:**
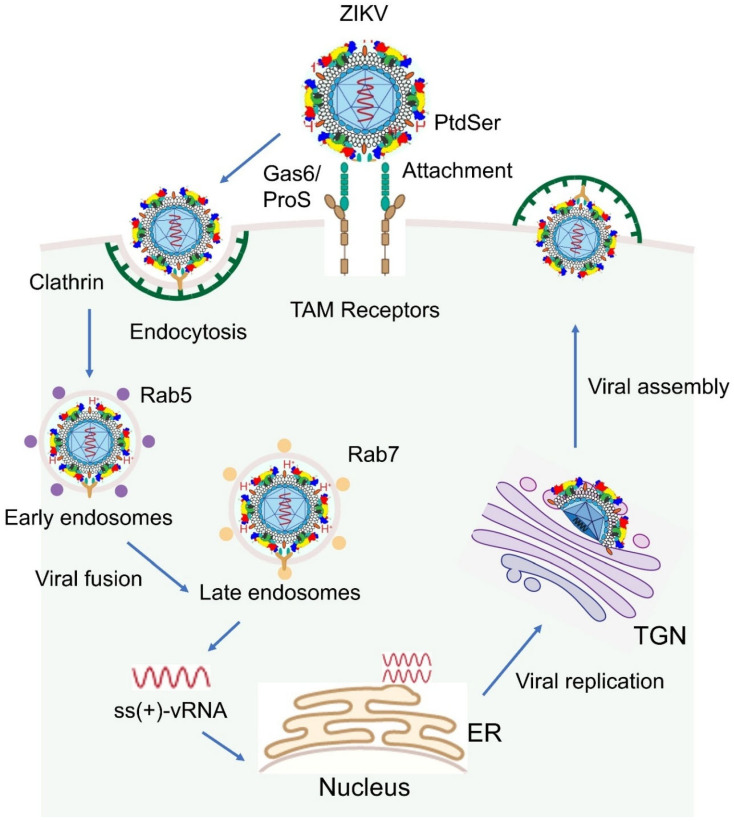
Schematic overview of the ZIKV life cycle. Upon contact with host cells, ZIKV initiates infection by binding to TAM family receptors on the host cell surface via the bridging molecules Growth arrest-specific 6 (Gas6) and Protein S (ProS), a process mediated by the viral E protein. Note that, besides TAM receptors, other receptors can also be used, which are described in the text. Following attachment, the virus enters host cells through clathrin-mediated endocytosis. Within endosomes, the acidic environment triggers fusion between the viral envelope and the endosomal membrane, leading to the release of the single-stranded, positive-sense viral RNA [ss (+) vRNA] into the cytoplasm. The viral RNA is immediately translated to produce viral proteins. Replication of the viral genome occurs on the ER surface, where double-stranded RNA (dsRNA) intermediates are synthesized and subsequently used to generate additional viral genomes. Immature virions are assembled in the ER, transported through the TGN for maturation, and finally released from the host cell as fully infectious particles. For indications of different color structures on ZIKV, please see Figure 1A.

To initiate infection, ZIKV first binds to host cell surface receptors, such as AXL, TYRO3, and MER, which are members of the TAM (TYRO3, AXL, and MER) receptor tyrosine kinase family, a process facilitated by the E protein (Figure 2). The virus then enters host cells via clathrin-mediated endocytosis, where it fuses with the endosomal membrane in a pH-dependent manner. This viral–host membrane fusion releases the viral genomic RNA into the cytoplasm. The +ssRNA of ZIKV acts directly as messenger RNA (mRNA), allowing immediate translation upon entering the host cell. The translation of the viral genome produces a polyprotein, which undergoes enzymatic cleavage to generate both structural and non-structural proteins as described above. Viral replication occurs on the surface of the endoplasmic reticulum (ER), where new viral RNA strands are synthesized. The viral RdRP synthesizes double-stranded RNA (dsRNA) from the positive-sense RNA genome. This dsRNA is then transcribed and replicated to produce additional viral genomes. Electron microscopy (EM) studies have shown that ZIKV replication is confined to the ER compartment in astroglial cells and neurons, indicating membrane-associated viral replication within the CNS [35]. During pregnancy, ZIKV replicates in the placenta and fetal brain, particularly in the Hofbauer cells of the placenta [36], which can serve as a reservoir for viral replication and dissemination of the virus to the fetus, leading to potential complications like microcephaly. Immature virus particles are assembled in the ER and then pass through the TGN, where they mature fully. Mature virions bud from the host cell membrane, acquiring a new viral envelope before being released into the extracellular environment [1,37]. Once released, these new viral particles can infect neighboring cells, initiating another round of the infectious cycle [38].

## 2. ZIKV Envelope Protein

ZIKV E protein is the primary structural component present on the surface of the viral particle, forming a layer of rafts composed of three parallel E protein dimers. This protein plays a pivotal role in the viral life cycle, facilitating viral binding to host cell receptors, mediating membrane fusion during viral entry, and interacting with the host’s innate and adaptive immune responses. The E protein is crucial for the virus’s ability to infect cells and trigger pathogenic effects [1,20,39,40,41].

### 2.1. The Structure of ZIKV E Protein

**Figure 3:**
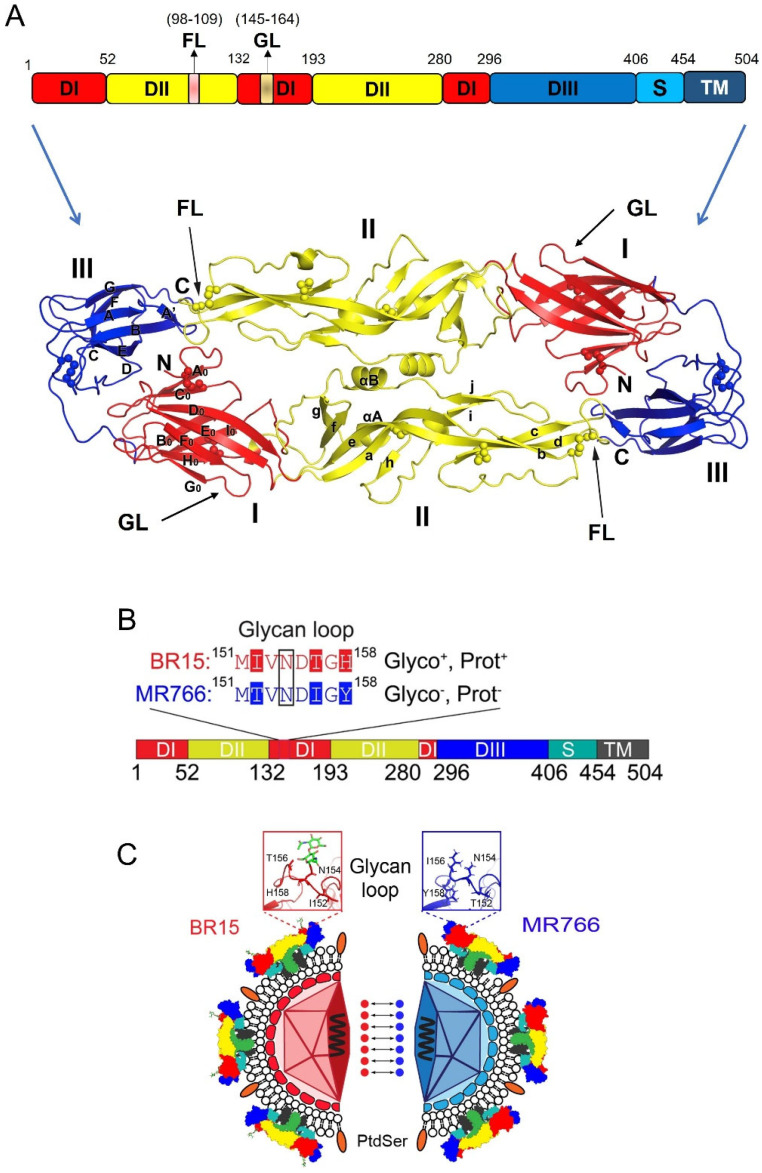
ZIKV E protein and its distinctive structural features between MR766 and BR15 strains. (**A**) Schematic representation of monomer sequence (**top**) and 3-D dimer (**bottom**) of the ZIKV E protein. Structural organization of the E protein monomer, which consists of three N-terminal ectodomains—Domain I (DI), Domain II (DII), and Domain III (DIII)—followed by a C-terminal stem-transmembrane (TM) region that anchors the protein to the viral membrane. Key structural features such as the fusion loop (FL) and glycan loop (GL), along with their respective residue positions, are indicated (**bottom**). Three-dimensional structure of the E protein dimer, highlighting DI, DII, DIII, and the FL. The 3-D E protein structure is adapted from [44]. (**B**) Organizational sequence of the E protein monomer shows differences in glycosylation and protonation status between BR15 and MR766. (**C**) Mirror image comparison of BR15 and MR766 E proteins, highlighting structural differences in the GL. Seven dot connections mark the evolutionarily distinctive a.a. residues within the GL region. The GL shown in the two square boxes illustrates the structural differences between BR15 (red) and MR766 (blue). For indications of different color structures on ZIKV, please see Figure 1A. Abbreviations: Glyco, glycosylation; Prot, protonation state; FL, fusion loop.

The E protein exists as a dimer on the surface of the mature virion ZIKV. The monomer of E protein is composed of 504 amino acids (a.a.) and has a molecular weight of approximately 54 kDa. Structurally, it is divided into four key domains: three N-terminal ectodomains (DI, DII, and DIII; 1–406 a.a.) and a C-terminal stem-transmembrane (TM) domain (407–504 a.a.), which anchors the protein to the viral membrane (Figure 3A) [40]. The ectodomains (DI, DII, and DIII) form a β-strand-rich surface that is involved in interactions with the host cell membrane and immune system components [40,42,43,44].

Domain I (DI) consists of 132 residues, and it serves as a link to connect DII and DIII, acting as a hinge region that allows flexibility in the protein structure (Figure 3A). DI can be further divided into three segments: the N-terminal region (1–52 a.a.), the central portion (132–193 a.a.), and the C-terminal region (280–296 a.a.) [40,44]. A notable feature within DI is the glycan loop (GL; a.a. 145–164), a flexible structure that can undergo post-translational N-glycosylation at the specific asparagine (N154) residue, potentially influencing viral infectivity [45,46]. Domain II (DII) has a distinctive finger-like structure and is divided into two segments (a.a. 53–131 and a.a. 194–279). It contains a pH-sensitive fusion loop (FL; a.a. 98–109), which is a critical element for the merging of the viral membrane with the host cell membrane during viral entry [44,47]. Domain III (DIII), spanning residues 297–406, plays a crucial role in binding of host cell receptors [41,48,49,50]. DIII is also a site where several neutralizing antibodies bind, conferring protection against ZIKV infection [51,52]. Moreover, DIII contains an extended CD-loop (a.a. 340–360), which is critical for viral stability and pathogenicity. Disruptive mutations or shortening of the CD-loop destabilize the virus, resulting in significantly reduced viral infectivity and attenuation [53]. This makes the CD-loop an important target for the development of neutralizing antibodies (nAbs) and live attenuated vaccines against ZIKV.

### 2.2. Post-Translational Modification of ZIKV E Protein

#### 2.2.1. Glycosylation of ZIKV E Protein

The ZIKV E protein can undergo N154 glycosylation, where an N-glycan is attached to the amide nitrogen of the N residue through post-translational modification (Figure 3B) [54,55]. However, the occurrence of glycosylation at the N154 residue of ZIKV E protein depends on several factors. For instance, the presence of serine (S) or threonine (T) at position 156 (154N-X-S/T156, X denotes any residue) is required for glycosylation to take place [20,56]. Glycosylation of the E protein is a critical factor in facilitating viral entry into host cells by interacting with host cell receptors, making it a significant contributor to ZIKV pathogenesis [56]. Additionally, the residues surrounding the N154 glycosylation site, specifically positions at 152, 156, and 158, have been shown to influence conformational changes in the E protein in response to intracellular pH changes [57]. This suggests that the glycosylation status at N154, potentially influenced by these surrounding residues, may also play a role in E-mediated membrane fusion during viral entry. ZIKV E protein exhibits considerable N-glycan heterogeneity across insects such as mosquitos and mammalian cell types, including monocytes, placental, and neural cells, consistent with patterns observed in DENV E protein [45,58]. Routhu et al. showed that the ZIKV E protein displays high heterogeneity N-glycans—from high-mannose (e.g., Man5GlcNAc2) to highly sialylated complex types—depending on the producing cell type. This glycosylation variability is influenced by both viral strain and host cell physiology, potentially impacting ZIKV pathogenesis [45]. Another study confirmed through enzymatic de-glycosylation that the N444 (E154) site in the ZIKV E protein is highly glycosylated and exhibits considerable micro-heterogeneity from the ZIKV E protein produced in EB66 cells [59]. Additionally, early studies suggest that N154 glycosylation patterns in ZIKV can differ significantly depending on whether the virus is derived from mosquitoes or human cells. For example, a study on the molecular evolution of ZIKV proposed that the loss of the N154 glycosylation site in the E protein may represent an adaptive response to the mosquito vector *Aedes dalzieli* [31]. In another study, genome-wide mapping of functional residues in the ZIKV E protein using a mutant library identified N154 glycosylation as the most divergent determinant of viral replication fitness between mosquito and human cells. This finding highlights the critical role of N154 glycosylation in modulating ZIKV replication efficiency across host cell types [60].

#### 2.2.2. Protonation of ZIKV E Protein

Protein protonation refers to the process of adding a hydrogen ion (proton, H^+^) to a specific a.a., such as histidine, which introduces a positive charge to the residue. This can significantly affect the protein’s structure and function, depending on the pH of the surrounding environment. The ZIKV E protein contains several potential protonation sites at H158, H249, H288, H323, and H158 [57,61]. Among them, H158 is particularly significant because it resides within the GL of the ectodomain DI (Figure 3B) [57]. During the initial membrane fusion between the virus and the endosomes, the low-pH environment is thought to trigger protonation at H158. This likely induces conformational changes in the GL, disrupting the E protein rafts on the virus surface and leading to the formation of fusion pores that allow the nucleocapsid to be released into the cytosol [57]. However, this mechanism remains to be thoroughly investigated.

#### 2.2.3. Phosphatidylserine on ZIKV E Protein

Phosphatidylserine (PtdSer) is a phospholipid present on the viral envelope of ZIKV and other enveloped viruses, playing a vital role in the viral infection process [62,63]. Findings showed that PtdSer in flaviviruses like DENV normally embedded within the lipid bilayer; PtdSer is interlocked with E proteins to form “raft-like” structures on the viral surface, which help maintain viral stability and function [64]. Under certain conditions, such as specific cell surface receptor signaling, PtdSer can be exposed on the outer surface of the virus [65]. This “flip” of PtdSer from the inner to the outer surface, mediated by scramblases [66], allows the virus to act as a “molecular mimic” of apoptotic cells. This mimicry enables the virus to bind to host cell receptors like AXL through the protein Gas6, facilitating viral entry (Figure 2) [62,67]. Research has shown that disrupting PtdSer on the ZIKV envelope significantly reduces the virus’s ability to infect cells [63], underscoring the importance of PtdSer in the viral entry process. However, the precise mechanisms behind this interaction remain underexplored.

#### 2.2.4. Consideration of Epistatic Interaction

Epistatic interactions within ZIKV E protein could also play a crucial role in its function and evolution. Studies have shown that mutations at one site in the E protein can affect the phenotypic consequences of mutations at other sites, influencing traits such as antibody recognition and viral entry [68,69]. As such, mutations in the E protein can disrupt its interaction with prM, leading to a misfolded or non-functional E protein, which then affects its ability to interact with host cell [70]. Another study provided a comprehensive map of how amino acid mutations in the E protein influence viral replication in a permissive cell line and enable escape from two monoclonal antibodies, ZKA64 and ZKA185 [71]. Additional investigation is needed to substantiate the epistatic interactions with ZIKV E protein.

### 2.3. Distinctive Features of E Proteins Between Ancestral African and Epidemic ZIKV

**Figure 4:**
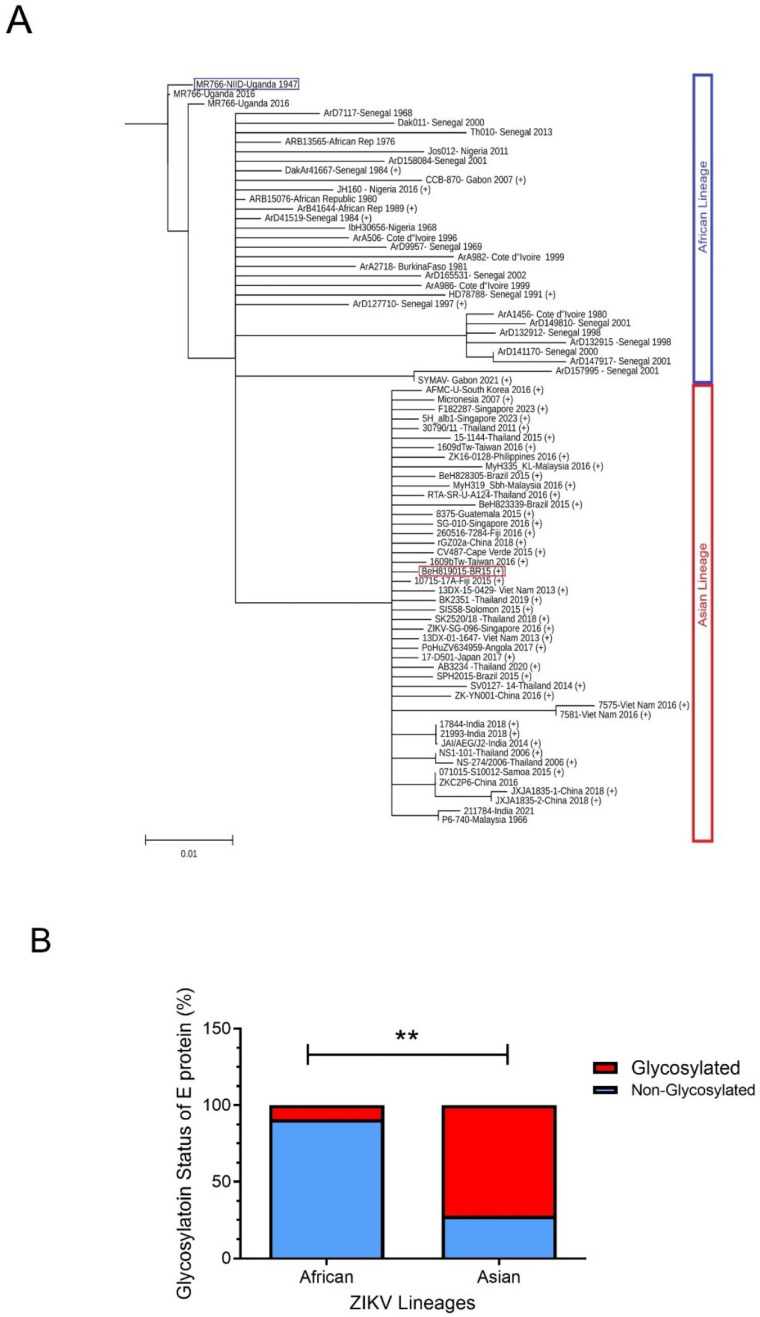
Phylogenetic differences in ZIKV E proteins between African and Asian lineages. (**A**) Phylogenetic tree analysis of ZIKV E protein sequences derived from available database entries. The tree depicts evolutionary relationships with a specific focus on a.a. residue 156, a key determinant of glycosylation at residue N154. Strains encoding serine (S) or threonine (T) at position 156 are predicted to be glycosylated at N154 [20,56], indicated by a “+” symbol (Gly+), whereas those with isoleucine (I) or other residues at this position are considered non-glycosylated. Two representative strains, BeH8190-BR15 (marked with a red square) and MR766-NIID-Uganda 1947 (marked with a blue square), are highlighted for detailed comparison in the main text. The phylogenetic tree was generated using the neighbor-joining (NJ) and maximum-likelihood (ML) methods, with bootstrap analysis contingent on 1000 replicates based on multiple sequence alignment of ZIKV E proteins using Clustal Omega version 1.2.3. [76]. The unique and complete ZIKV E sequences available in GenBank up to May 2024 were retrieved for this analysis. The resulting trees were visualized using TreeViewer v2.2.0 [77]. The scale bar represents a genetic distance of 0.01, indicating 1% sequence divergence among the aligned E protein sequences. (**B**) Comparison of the relative abundance of glycosylated (**red**) versus non-glycosylated (**blue**) E proteins across lineages. The N-linked glycosylation site at position 154 of the E protein was analyzed among ZIKV strains with unique E protein a.a. sequences, which were obtained from the GenBank virus genome database (http://www.ncbi.nlm.nih.gov/genome/viruses/, accessed on 15 March 2025), Among African lineage viruses, the majority (90.9%; *n* = 44) lack glycosylation at N154, whereas most Asian lineage viruses (72.3%; *n* = 36) are glycosylated at this position. Red box, glycosylated; blue box, non-glycosylated. **. Statistical significance, *p* < 0.005.

ZIKV can be divided into two major lineages based on their geographic origins and genetic differences [72]. The African lineage is considered the ancestral viruses, while the Asian lineage has been responsible for recent major outbreaks in the Pacific and Americas. Generally, the Asian lineage is considered more concerning due to its potential for causing severe complications like microcephaly when transmitted during pregnancy, while the African lineage typically leads to milder symptoms in humans. Conversely, in animal models, African strains are generally considered more virulent, causing more severe infections and higher mortality rates compared to Asian strains, which tend to be milder [73,74]. Genetic differences exist between these two lineages, particularly in the viral proteins, which contribute to the observed differences in virulence, which can be demonstrated by their evolutionary distance and by comparing their respective viral genomes using phylogenetic tree analysis [75]. Indeed, phylogenetic trees generated by comparing E protein sequences (Figure 4A) also show similar and distinctive genetic distances between African and Asian ZIKV lineages, similar to those phylogenetic trees generated based on the entire viral genomes.

Careful examination of the sequence alignments of E proteins reveals some distinctive features that can separate E proteins of ancestral African lineages from those of epidemic Asian ZIKV viruses. To aid in easy comprehension, here we use a pair of ancestral (MR766; GenBank accession#: LC002520) and epidemic (BR15, *aka* BeH819015; GenBank accession#: KU365778) viral sequences to illustrate those differences in E proteins between African and Asian ZIKV lineages (Figure 3B,C). These two viral sequences are chosen as representatives of these two types of viruses because the MR766 ZIKV strain was the first ever reported ZIKV, isolated in 1947 from Africa [5], and BR15, isolated in 2015 from the blood of an epidemic patient in the Pará state [78]. Besides their differences in historic timeline and human disease-causing status [79,80], these two viruses are chosen because, virologically, these two viruses show distinctive contrasts in their ability to bind, infect, replicate, and propagate in neurotropic cells [27,57,81]. For example, testing of chimeric viruses between these two strains by swapping structural proteins suggests that the E protein plays a prominent role in viral binding [27,57,81,82].

Evolutionary analysis with protein sequence alignments of the ancestral MR766 and the epidemic BR15 E protein sequences reveals that BR15 has 15 distinctive a.a. compared to MR766 [81,83]. Of these, seven are evolutionarily distinctive (Figure 3C): three in DI (T152I, I156T, and Y158H) and four in DIII (V317I, I341V, V343A, and D293E), with the three underlined a.a. being human-specific adaptations [81,83]. Notably, all three unique mutations (T152I, I156T, and Y158H) in the DI domain of the epidemic BR15 ZIKV protein are within the GL, which contains both an N-glycosylation site at N154 and a protonation site at H158 (Figure 3C) [84]. In contrast, the ancestral ZIKV MR766 lacks both the N154 glycosylation and the H158 protonation site. The ZIKV E GL extends towards the DII FL and modulates the conformational dynamics of the virion [84], suggesting that the differences in the GL between these two viruses may contribute to differences during viral entry. Most significantly, based on analysis of E protein sequences deposited in GenBank, the majority (90.9%) of epidemic ZIKV E proteins, including BR15, are N154-glycosylated, as they contain an S156 or T156 residue. In contrast, most African isolates (72.3%), including MR766, lack this glycosylation site, and therefore have non-glycosylated E proteins (Figure 4B) [56,85]. The functional importance of E glycosylation and protonation will be further discussed in the following sections.

## 3. Viral Entry Mediated by ZIKV E Protein

### 3.1. The Cellular Receptors

The ZIKV E protein mediates viral attachment and fusion with host cell membranes during viral entry. To initiate specific viral attachment and fusion, the ZIKV E protein interacts with multiple cell receptors, including AXL, TYRO3, MER, TIM-1, and DC-SIGN, allowing the virus to efficiently infect a wider range of host cells by providing multiple entry points [86,87,88]. For example, AXL has been identified as a key cell surface receptor facilitating ZIKV entry across various cell types [89,90,91,92]. During viral entry, the ZIKV E protein interacts with the host cell receptor AXL through a bridging molecule called Gas6. Gas6 binds to the viral membrane’s PtdSer, effectively connecting the virus to the AXL receptor on the cell surface and facilitating viral entry. Although ProS1 (Protein S) may play a minor role in this interaction, its contribution is generally considered less significant compared to Gas6 in the context of ZIKV infection (Figure 2) [93,94].

By utilizing AXL as a receptor, the ZIKV E protein can efficiently target specific brain cells, particularly neural progenitor cells, which express high levels of AXL, contributing to the virus’s neuropathogenesis. For instance, Espino et al. showed the critical role of AXL for ZIKV entry in decidual stromal cells and revealed that blocking the interaction between the ligand–receptor during the initial infection stage significantly reduces virus pathogenesis at the maternal–fetal interface [95]. However, AXL is not the only receptor used during infection in the brain. Studies have shown that even when AXL is inhibited or knocked out, ZIKV can still infect certain brain cells, indicating that alternative receptors may be involved [91,96]. Indeed, research suggests that other TAM receptors such as TYRO3 and MER also play a role in ZIKV infection, albeit to a lesser extent than AXL [97,98]. All of them bind to Gas6 and ProS1 ligands for TAM receptor activation (Figure 2) [93,94]. Furthermore, blocking both AXL and TIM-1 with antibodies can significantly inhibit ZIKV infection, indicating that both receptors may work together to facilitate viral entry [97]. TIM-1, like other TAM family receptors, also binds to PtdSer, facilitating viral adsorption and internalization. However, research indicates that while TIM-1 plays a role, the primary receptor for ZIKV E is AXL, with TIM-1 acting as a secondary or supporting receptor [99]. Therefore, the specific receptors used by the ZIKV E protein depend on the type of cell being infected [100]. An example of receptor-mediated susceptibility is the DC-SIGN receptor, whereby the absence of DC-SIGN on Langerhans cells renders them non-permissive to Zika virus infection, in contrast to DC-SIGN–expressing dendritic cells that support viral entry and replication [101].

### 3.2. ZIKV E Protein-Mediated Virus Attachment to Host Cells

#### 3.2.1. The Importance of E Protein Structure

The ZIKV E protein is essential for viral attachment to host cells, and any structural modifications can significantly impact viral entry, infectivity, and cell tropism [102]. The DIII of the E protein plays a crucial role in receptor binding [41,48,49,50], with specific mutations such as A310E and E393K altering cell entry and tropism [102]. Additionally, the CD-loop in DIII is vital for maintaining viral stability, as mutations or shortening of this loop lead to destabilization, reducing infectivity and attenuating the virus [53]. Similarly, a synthesized peptide containing residues 268–273 in DII of the E protein effectively competes with viral attachment and disrupts infection, highlighting the critical role of DII in viral attachment and infectivity [44,70,103].

#### 3.2.2. The Role of N154 Glycosylation

Post-translational modifications of the ZIKV E protein, such as glycosylation, play a crucial role in its interaction with cell surface receptors, influencing viral tropism and infectivity [104]. The ZIKV E protein possesses a well-characterized N-linked glycosylation site at a.a. 154, a highly conserved feature among orthoflaviviruses. This glycan extends from the viral surface and facilitates receptor binding. For instance, glycosylation at N154 enhances ZIKV infection in DC-SIGN-expressing human cells, suggesting its role in receptor interaction [101]. Additionally, viruses retaining this glycosylation site exhibit heightened pathogenicity in mouse models [56,85]. Conversely, viruses lacking glycosylation or those carrying the N154Q glycosylation-deficient mutation display attenuated virulence in mice, leading to lower viral loads in serum and brain tissues [56,85,105]. Interestingly, immunization with the N154Q mutant virus provided protection against wild-type ZIKV (Cambodian strain FSS13025), indicating its ability to elicit a strong antibody response [105].

Studies using pseudoviral particles further confirm the importance of N154 glycosylation, showing a reduction in infectivity when this glycan is absent [106]. Moreover, non-glycosylated viruses replicate inefficiently in mouse brains compared to their glycosylated counterparts, reinforcing the role of N154 glycosylation in ZIKV neurovirulence and invasion [85]. Interestingly, the N-glycans associated with the ZIKV E protein vary in structure and composition depending on viral strain and host cell type [45]. Inhibition studies using mannose-rich competitive inhibitors demonstrated a significant reduction in ZIKV infection in DC-SIGN-expressing cells, suggesting that high-mannose N154 glycans are crucial for interaction with C-type lectin receptors such as DC-SIGN [45]. However, the specific impact of those heterogeneous N-glycans on N154 glycosylated viruses during viral entry, infectivity, and cell tropism has yet been fully investigated. Our own study shows the difference between MR766 and BR15/ICD in viral binding and subsequent viral infection and replication, as well as apoptotic cell death [27,81], suggesting N154 glycosylation of the E protein may play a critical role in viral attachment. However, the molecular action underlying how N154 glycosylation affects the viral binding remains unclear.

The E glycosylation appears to be critical for ZIKV infection of mammalian and mosquito cells, because a glycosylation mutant N154Q diminished oral infectivity by *Ae. aegypti* vector and showed reduced viremia and diminished mortality in mouse models [105]. While non-glycosylated ZIKV displayed reduced neuroinvasion when introduced subcutaneously, it replicated efficiently following intracranial inoculation, implying a role for E glycosylation in BBB penetration [85]. Furthermore, ZIKV viral particles lacking the E protein glycan were still able to infect Raji cells expressing the lectin DC-SIGN receptor, indicating the prM glycan of partially mature particles can facilitate the viral entry [84]. The E protein, specifically its extended CD-loop, may confer viral stability, cell cycle-dependent viral replication, and in vivo pathogenesis, as shortening the CD-loop compromise the virus, and Δ346 mutation in this loop disrupts thermal stability of the virus [53].

#### 3.2.3. The Role of PtdSer

PtdSer, a phospholipid primarily found on the inner leaflet of cell membranes on the viral envelope, has been implicated in facilitating the binding of enveloped viruses [63]. Since the phospholipid PtdSer is interlocked with E proteins to form “raft-like” structures on the virus surface and serves as a receptor-binding ligand, it may also play a role in viral attachment and viral entry [62,64,107]. Limited studies have shown that PtdSer indeed support viral entry and ZIKV infection in human and mosquito cells as well as in a mouse model [63]. Furthermore, a PtdSer-specific inhibitor could reduce the viral load in serum and spleen in the mouse model [63]. One interesting feature of PtdSer, observed in DENV, is its exposure to the outer viral surface upon an increase in temperature. For example, at a low temperature of 4–28 °C, orthoflavivirus virions display a “closed smooth surface morphology” where PtdSer are hidden behind the viral envelope. Thus, they are less efficient in attaching to host cells. In contrast, when the temperature is shifted to physiologically relevant human body temperature of 37 °C, virions show a “bumpy morphology” where PtdSer are extended out from the viral envelope. As a result, they bind to host cells with high efficiency [108]. As glycosylated and non-glycosylated ZIKV binds to host cells differently, besides N154 E glycosylation of E protein, another possibility to explain the observed differences in viral entry, infectivity, and cell tropism could be the phospholipid variation on the virus surface. However, no research has yet been done in this regard.

### 3.3. The Role of ZIKV E Protein in Endocytosis and Viral Fusion

Following E protein-mediated viral attachment to host cells, ZIKV enters host cells primarily through clathrin-dependent endocytosis (CDE) (Figure 2) [1,109], although clathrin-independent endocytosis (CIE) might also occur [109]. After the virus is internalized *via* clathrin-coated pits, it is enclosed in an endocytic vesicle and transported to early Rab5+ endosomes (pH 6.8–6.3), where it initiates infection. As the endosome matures into a late Rab7+ compartment and the pH declines to approximately 6.2–5.0, the E protein undergoes a pH-dependent conformational shift. This exposes its FL, facilitating the fusion of the viral envelope with the endosomal membrane. Consequently, the viral RNA genome is released into the host cell cytoplasm within 10 to 15 min of infection [110,111].

A comparative study of viral entry between the African and Asian ZIKV lineages reveals that the clathrin-dependent entry mechanism is highly conserved across both lineages [111]. ZIKV predominantly fuses with late endosomes, likely due to the low pH in these endosomes inducing a conformational change in the ZIKV E protein, which facilitates its interaction with the host cell membrane and subsequent fusion [111]. The E-152/156/158 residues of the ZIKV E protein are believed to be crucial in the later stages of viral entry, particularly in facilitating the conformational changes required for membrane fusion with the host cell [57]. This may be due to their location within the GL of the E protein, where notable differences in N154 glycosylation and H158 protonation status exist between the African and Asian lineages (Figure 3B,C).

## 4. ZIKV E Protein-Mediated Immune Response and Evasion

The protective immune response serves as a first-line defense of the cellular immune system against viral infections, typically initiated by the release of cytokines and chemokines. As the major surface protein of ZIKV, the E protein functions as the principal viral antigen, eliciting host innate and adaptive immune responses [44]. Notably, N154 glycosylation of the E protein plays a critical role in modulating interactions with the host immune system.

A study investigating ZIKV infection in human-induced pluripotent stem cell (hiPSC)-derived macrophages and microglia found that an epidemic Brazilian ZIKV strain (GZ01/2016; KU820898) carrying a glycosylated N154 E protein triggered a stronger immune response than the non-glycosylated MR766 strain. The glycosylated strain induced higher levels of immune mediators, including AIF1, IL-6, IL-1β, IL-10, G-CSF, and CCR5 [87]. Furthermore, the expression of AXL and TYRO3, both TAM receptors, was significantly elevated in cells infected with the epidemic strain compared to MR766, suggesting that N154 glycosylation enhances viral attachment and immune activation [87].

Interestingly, a contrasting effect of E protein glycosylation was observed in primary mouse dendritic cells, where N154 glycosylation led to a weaker immune response. The glycosylated virus elicited lower levels of IFN-α, IFN-β, IL-1β, and IL-6 compared to the non-glycosylated N154Q mutant [105]. The same study also demonstrated that E protein glycosylation negatively regulated viral attachment, virion assembly, and progeny infectivity in the mosquito C6/36 cell line [105].

These discrepancies may arise from differences in host cell receptor expression and immune responses that influence viral replication. They also suggest that the impact of E protein glycosylation is host- or context-dependent. For example, glycosylation may influence viral interactions with different receptors or modulate distinct cellular pathways, leading to variable outcomes depending on the host species or cell type involved. Another possible explanation is that the role of E protein glycosylation depends on the specific cell surface receptors engaged during infection. In the human macrophage study, TAM receptors were prominently upregulated [87], whereas in mouse dendritic cells, ZIKV primarily utilized DC-SIGN as the entry receptor [105].

Supporting this notion, our recent data show that the epidemic BR15 strain, which carries a glycosylated N154 E protein, induced higher immune responses than the non-glycosylated MR766 strain in the neuroblastoma SH-SY5Y cell line. Similar to the findings of Mesci et al., BR15 triggered elevated levels of IL-6, IL-1β, IL-10, and CCR5 [87]. Additionally, we observed activation of other proinflammatory cytokines, including IL-8, CCL2 (MCP-1), and CCL5 (RANTES) (our unpublished data). However, in contrast to Mesci’s findings, our results indicate that the N154-glycosylated BR15 virus exhibited reduced viral binding, replication, and apoptotic cell death compared to the non-glycosylated MR766 strain. Although other viral factors may contribute to these differences, receptor expression variations between cell types could potentially explain these discrepancies. Unlike hiPSC-derived macrophages and microglia, which express AXL and TYRO3, SH-SY5Y cells predominantly express AXL and MER [112]. These differences in receptor engagement may influence the extent of immune activation and viral replication, highlighting the complexity of E protein glycosylation in ZIKV pathogenesis.

One potential argument against using the MR766 strain for E glycosylation studies is that this strain has been passaged too many times in vitro, potentially leading to non-representative results [79]. However, the only difference between the glycosylated and non-glycosylated viruses in the Fontes-Garfias’ study [105] was the N154Q mutation in the E protein, underscoring the importance of E glycosylation in these findings. Additionally, the role of N154-glycosylation in eliciting host immune responses is supported by numerous clinical reports. Similar proinflammatory cytokines like IL-1β, IL-6, IL-10, and G-CSF were elevated in the blood, brain, cerebrospinal fluid, and amniotic fluid of ZIKV-positive pregnant women [113,114,115,116]. Lastly, animals inoculated with recombinant ZIKV E protein alone also elicit various protective and proinflammatory cytokines, including IFNγ, TNFα, IL-4, IL-6, and IL-12 [117,118,119].

Besides triggering host cellular inflammatory immune responses as described above, ZIKV also activates the production of Type I interferon (IFN) as part of the host antiviral response [1,120]. This antiviral IFN response is facilitated by mechanisms involving the activation of Toll-like receptors (TLRs), particularly TLR3 and TLR7/8, as well as the RIG-I-like receptor (RIG-1) [1,120]. TLR3- and RIG-1-mediated Type I IFN production, followed by activation of the JAK/STAT pathway, help the host cell mount resistance against ZIKV infection [121].

During the initial stages of infection, ZIKV interacts with host cell surface receptors such as AXL, which not only promotes viral entry but also suppresses host Type I IFN response. This occurs through the interaction of ZIKV E protein with Gas6, triggering the AXL signaling pathway and allowing immune evasion by suppressing the antiviral response [91,122]. The role of ZIKV E protein in immune evasion has been demonstrated through experiments using the human glial cell line SNB19, where Atranorin, a secondary metabolite of lichens, inhibited ZIKV infection by attenuating the activation of the IFN signaling pathway through direct targeting of the E protein [123].

In addition to evading IFN responses, ZIKV infection activates the host complement system, a key component of both the innate and adaptive immune systems. The complement system functions to defend against viral infections by forming the membrane attack complex (MAC), which creates pores in the viral membrane, leading to virolysis or complement-mediated lysis [116,124]. ZIKV has developed mechanisms to counteract the complement system. One study found that ZIKV E protein interferes with the formation of the MAC by binding to terminal complement proteins like C9, reducing polymerization and thereby preventing effective complement-mediated virolysis [125]. This direct interaction with the complement system allows ZIKV to evade destruction by the host immune response.

N154 glycosylation of the E protein may also enhance viral infection by aiding in the evasion of the host’s innate antiviral response. For instance, the glycosylated E protein inhibits the reactive oxygen species (ROS) pathway, which is essential for ZIKV infection in *Aedes* mosquitoes. Notably, mutating the glycosylation-promoting residue T156 to either A or I, thereby removing N154 glycosylation, prevents mosquito infection [126]. This phenotype can be rescued by inhibiting the ROS pathway, suggesting that the glycosylated E protein facilitates viral evasion of the mosquito’s innate immune defenses to enhance infection. Supporting this notion, *Aedes* mosquitoes infected with the N154-glycosylated Cambodian ZIKV strain FSS13025 exhibited significantly higher infection rates compared to those infected with the non-glycosylated N154Q mutant of the same virus [105]. Similarly, in an immune-compromised IFNAR^−/−^ A129 mouse model, E glycosylation substantially increased mortality, accompanied by high viremia. In contrast, the N154Q mutation markedly attenuated ZIKV, as evidenced by reduced viremia, minimal weight loss, and complete survival [105].

## 5. Neutralizing Antibodies, Antibody-Dependent Enhancement, and Vaccine Development

### 5.1. Neutralizing Antibodies (nAbs)

The nAbs are crucial components of the host immune response against ZIKV infection. The E protein, being the primary antigen on the surface of the virus, serves as a major target for nAbs. These antibodies typically prevent infection either by blocking the virus from binding to its host cell receptors or by inhibiting the structural changes required for the E protein to mediate fusion during viral entry via the endocytic pathway [127].

As described earlier, the ZIKV E protein consists of three structural domains: DI, DII, and DIII (Figure 3A). Domain I serves as a connector between DII and DIII, acting as a flexible hinge that enables the conformational changes necessary for viral entry into host cells [128]. While all three domains can be targeted by nAbs, DIII is particularly important because it is responsible for host cell receptor binding, making it the primary target of nAbs against the ZIKV [52,129]. Due to this critical role in neutralization, DIII is often a key focus in ZIKV vaccine development [52].

Numerous DIII-specific nAbs have been isolated from both convalescent patients and immunized mice (Table 1). Three distinct epitopes in the DIII domain have been identified: the lateral ridge (LR), the C–C′ loop, and the ABDE sheet region, which is a specific region that is highly conserved among different ZIKV sequences [130]. The LR is a prominent structural feature formed by loops connecting β-strands, while the C–C′ loop is a flexible region that plays a vital role in receptor binding and shows variability across ZIKV strains [131]. The ABDE sheet is a β-sheet region that acts as an important epitope for nAbs [70].

In early studies using X-ray crystallography and competition binding analyses, six mouse monoclonal antibodies (mAbs) against ZIKV were analyzed. Among these, ZV-48 and ZV-64 bind to the C–C′ loop, ZV-2 binds to the ABDE sheet, and ZV-54 and ZV-67 target the LR of the DIII domain. ZV-48, ZV-54, ZV-64, and ZV-67 were specific to ZIKV and neutralized all ZIKV lineages [132]. However, only ZV-67 neutralized all ZIKV strains in vitro and provided protection in lethal challenge models in vivo [132]. Other mAbs, such as ZIKV-116, also target the LR and prevent ZIKV infection by blocking viral fusion with host membranes [131]. However, ZIKV-116 cross-neutralizes certain strains of DENV1, with higher neutralizing activity observed against Asian ZIKV strains compared to African strains [131]. Additional mAbs, such as Z004, Z006, and Z021, are part of a set of recurrent antibodies that neutralize both ZIKV and DENV [133,134]. In a separate study, five DIII-specific nAbs (HA-12, 1C-11, 2F-8, 1D-9, and 1D-11) were isolated from ZIKV-infected patients, all of which neutralized the Asian strain of ZIKV. Among these, HA-12 and 2F-8 also neutralized American strains, i.e., ZIKV of Asian lineage found in the America. The most potent antibody, 2F-8, conferred complete protection in mice when administered in a single dose, either before or after exposure to a lethal ZIKV challenge [135].

Besides targeting DIII, the junction between DI and DIII is another important site for nAbs. This region forms a quaternary epitope accessible only when viral proteins are in their native conformation. Antibodies targeting this junction can effectively block viral entry and neutralize infection by disrupting the virus’s ability to fuse with host cells, potentially offering high specificity to ZIKV while minimizing cross-reactivity with related viruses [129]. For example, the nAb B11F, recovered from a convalescent patient, targets a novel epitope that spans both DI and DIII [136]. Additionally, four other nAbs (ZKA190, MZ1, MZ4, and MZ24) target the DI/DIII linker region of the ZIKV E protein. ZKA190 demonstrated high efficacy in vivo, preventing morbidity and mortality in ZIKV-infected mice, while MZ1, MZ4, and MZ24 exhibited cross-neutralizing activity against both ZIKV and DENV [137,138].

In addition to DI and DIII, quaternary epitopes in the DII domain of the ZIKV E protein are also targeted by nAbs [139]. The nAb ZKA185, for instance, targets DII and neutralizes both African and Asian ZIKV lineages [140].

The FL, located between the DII and DIII domains, is another important target for nAbs due to its role in viral fusion with host membranes. Since this region is conserved across orthoflaviviruses and overlaps with the prM binding site in immature particles, it presents a target for broad-spectrum nAbs [141]. One such antibody, 2A10G6, provided broad protection against ZIKV, DENV1-4, and WNV in mouse models and in vitro studies. Human-derived mAbs such as AZ1p and AZ6m, which target the ZIKV E protein FL, also demonstrated broad binding capacity and neutralization of ZIKV, YFV, and DENV [142].

The GL within DI of the ZIKV E protein is also a target for nAbs due to its role in modulating E protein conformational changes during viral fusion [57,84]. A recombinant ZIKV protein (E80) covering 80% of the N-terminal ectodomain-induced potent nAb responses protected mice from lethal challenges of the ZIKV infection [51]. Subsequently, five mAbs (3E8, 5F8, 5G3, 8A2, and 9C3) were isolated from E80-immunized mice, showing specific neutralization of Asian-lineage ZIKV strains but not African strains [143]. Among these, 5F8 conferred complete protection in a mouse model by inhibiting viral entry during the early post-attachment stage. The epitope recognized by 5F8 is highly conserved in ZIKV but varies between ZIKV and other orthoflaviviruses, possibly representing a new class of ZIKV-specific nAbs targeting the GL [143]. However, other studies have shown that certain mAbs targeting the GL, such as A11 and A42, exhibit broader activity against ZIKV and other orthoflaviviruses [144]. These findings underscore the importance of the GL as a key antigenic determinant [43,82].

Another study further emphasized the role of the GL in nAb-mediated ZIKV neutralization [82]. As previously described, the GL contains three evolutionarily distinctive residues, 152, 156, and 158, within the ZIKV E protein (E-152/156/158), which in most cases can distinguish the African lineage from the Asian lineage (Figure 3B). To investigate the role of N154 glycosylation and H158 protonation in nAb-mediated ZIKV neutralization, researchers replaced the E-152/156/158 residues of the BR15 strain with those from MR766 (I152T, T156I, and H158Y), resulting in a non-glycosylated virus in the BR15 backbone [82]. When this chimeric ZIKV was inoculated into adult BALB/c mice, it rapidly induced nAb production. Interestingly, while the nAbs effectively neutralized the MR766 strain, they failed to neutralize the BR15 clone. This suggests that the E-152/156/158 residues in the GL, or the N154 glycosylation, may influence nAb epitope accessibility, and that differences in N154 glycosylation between MR766 and BR15 may lead to variations in neutralization [82].

In a follow-up study [43], the role of the GL-residing E-152/156/158 residues as antigenic determinants was further tested. An oligopeptide consisting of a 20-mer (a.a. 145–164) from the GL, which carried the non-glycosylated MR766-derived residues E-T152/I156/Y158, was used to assess immunoreactivity against anti-GL Abs from the serum of the same chimeric ZIKV-challenged mice described earlier [82]. Results showed that the 20-mer with MR766’s E-T152/I156/Y158 residues reacted with anti-GL Abs from the BR15-E-T152/I156/Y158-challenged mice. However, the 20-mer carrying BR15-derived E-I152/T156/H158 did not react with the same serum, suggesting that anti-GL Abs induced by the chimeric virus were specific to MR766. Notably, changing residues T152 to I152 and I156 to T156 did not alter the immunoreactivity of the anti-GL sera; whereas, the substitution of Y158 with H158 (Y158H) abolished the reactivity to the anti-GL sera. Structural analysis of the E-Domain I, including both the wild-type 20-mer and mutant E-T152I and E-I156T 20-mers, suggested that residues E-152 and E-156 influence the conformation of the GL. This alteration in GL structure could affect the antigenic reactivity of Abs targeting this region. While these studies did not specifically evaluate the roles of N154 glycosylation or H158 protonation, the findings suggest that the E-152/156/158 residues are likely key antigenic determinants within the ZIKV GL region [43].

The use of nAbs that target multiple domains of the ZIKV E protein could maximize therapeutic potential and prevent viral escape [133,140]. For instance, human-derived nAbs Z23 and Z3L1, which target tertiary epitopes in DI, DII, and DIII, exhibit potent ZIKV-specific neutralization and offer post-exposure protection in mice [145]. Immunization with the E protein or its subdomains (DI/DII and DIII) has also been shown to induce high titers of E-specific antibodies that neutralize ZIKV [119].

Finally, The E dimer epitope (EDE) of the ZIKV E protein, a unique feature formed by the interaction of two E protein dimers on the viral surface, is another significant target for nAbs due to its high specificity and ability to block viral entry [146]. EDE-targeting antibodies, such as EDE1-C8, EDE1-C10, and EDE1-B10, which were initially raised against DENV, also exhibit cross-neutralizing activity against ZIKV [139,147]. In mice, a single dose of EDE1-B10 administered three days post-infection, prevented death and reduced ZIKV levels in the brain and testes [148]. Similarly, the human-derived nAb ZIKV-117, which targets EDE, significantly reduced tissue damage, placental and fetal infection, as well as mortality in pregnant and non-pregnant mice [149,150].

**Table 1:** List of nAbs against ZIKV envelope protein.

Neutralizing Antibody	Target Site	Specificity	Reference
ZV-2, ZV-48, ZV-54, ZV-64, ZV-67	DIII or DIII LR	ZIKV	[132,149]
ZIKV-116	DIII	ZIKV	[131,149]
Z004, Z006, Z021	DIII LR	ZIKV and DENV1	[134]
HA-12, 1C-11, 2F-8, 1D-9, 1D-11	DIII	ZIKV Asian lineage	[135]
B11F	DI and DIII	ZIKV	[136]
ZKA190	DI-DIII linker, DIII LR	ZIKV	[137]
MZ1/MZ4/MZ24	DI/DIII linker region	ZIKV, DENV1-4	[138]
ZKA185	DII	ZIKV	[140]
2A10G6	DII (FL)	ZIKV, DENV1-4 and WNV	[44,141]
AZ1p, AZ6m	DII(FL)	ZIKV, YFV, and DENV	[142]
3E8, 5F8, 5G3, 8A2, 9C3	Linear Epitope on GL	ZIKV Asian lineage	[143]
A11, A42	GL	ZIKV	[144]
Z3L1, Z23, Z20	DI, DII, or DIII	ZIKV	[145]
rhMZ—Group A, B, C, D	EDE	ZIKV	[146]
EDE1-B10, EDE1-C8, EDE1-C10	EDE	ZIKV and DENV 1-4	[69,139,147]
ZIKV-117	EDE	ZIKV	[149,150]

### 5.2. Antibody-Dependent Enhancement (ADE) and Vaccine Development

ADE presents a significant challenge in the development of effective therapies against ZIKV infection. ADE occurs when non-neutralizing or sub-neutralizing levels of nAbs bind to ZIKV but fail to block the virus from infecting host cells. Instead, the antibody–virus complex facilitates viral entry into cells via Fc receptor-mediated internalization, leading to increased rates of infection and potentially more severe disease outcomes [151,152]. This phenomenon is particularly concerning in regions where both ZIKV and DENV co-circulate, as cross-reactive antibodies generated during primary DENV infection may enhance ZIKV infection upon secondary exposure, exacerbating disease severity [153,154]. Therefore, understanding ADE is essential for developing safe and effective vaccines against ZIKV infection [155].

Note that the occurrence of ADE in ZIKV infection remains an area of active investigation. Only limited clinical evidence suggests that ADE may occur, particularly in the context of pre-existing immunity to DENV. For instance, pre-existing DENV antibodies have been shown to influence the antibody response during primary ZIKV infection, leading to enhancement of ZIKV infection in vitro and in vivo [156,157]. An epidemiological study also found that convalescent sera from dengue patients exhibited ADE for ZIKV [158]. Conversely, a fatal case of DENV following prior ZIKV infection has been interpreted as a clinical example of ADE [159]. In addition to these clinical observations, several experimental studies have demonstrated that DENV-immune sera or monoclonal antibodies can enhance ZIKV infection through Fc receptor-mediated uptake in cell cultures and animal models [160,161]. Although the extent of ADE in natural ZIKV infection remains uncertain, these findings collectively support the possibility that ADE may occur in ZIKV under certain immunological conditions, especially in flavivirus-endemic regions.

As ZIKV E protein is a primary target for nAbs, many studies have focused on the immune responses elicited by this protein, including cross-reactivity with antibodies against other orthoflaviviruses such as DENV [44,162]. ZIKV and DENV are closely related viruses within the flavivirus family, sharing similar modes of transmission, clinical symptoms, and mosquito vectors, particularly *Aedes aegypti* [163]. DENV is the most prevalent orthoflavivirus in South America and represents the leading cause of orthoflavivirus-related diseases in Brazil [164]. However, the rising global temperatures have contributed to the spread of DENV, increasing its threat to public health in other regions, including the United States [165]. For instance, the first case of fetal DENV infection was reported in the U.S. in 2021, where the child had prior exposure to ZIKV, an outcome attributed to ADE [159]. Due to the high degree of similarity between ZIKV and DENV, about 55% a.a. identity in the E protein, ADE frequently arises from cross-reactive nAbs generated during a primary DENV infection, leading to an enhanced risk of secondary infection with ZIKV [166,167].

One approach to mitigating the risk of ADE in vaccine development is to identify ZIKV-specific nAbs that do not cross-react with other orthoflaviviruses, particularly DENV. For example, to minimize cross-reactivity between ZIKV and DENV, one study isolated four groups of EDE-specific nAbs from ZIKV-infected rhesus macaques. These antibodies not only protected against ZIKV replication in mice but were also identified in convalescent humans following ZIKV infection [146]. Moreover, a study showed that plasma from individuals previously infected with DENV could enhance ZIKV infection in human myeloid cells via ADE [168]. The mathematical modeling study also revealed that dengue vaccination, especially with imperfect pre-vaccination screening, can enhance ZIKV transmission through ADE, underscoring the need for cautious implementation in regions where both viruses co-circulate [169].

In another study, SIgN-3C, a human-derived antibody initially raised against DENV1-4, also neutralized ZIKV infection in an IFNAR^−/−^ mouse model [170,171]. However, a variant of this antibody, SIgN-3C-LALA, did not induce ADE in vitro and provided similar protection in vivo [171]. In pregnant ZIKV-infected IFNAR^−/−^ mice, treatment with SIgN-3C or SIgN-3C-LALA significantly reduced viral loads in fetal organs and placenta, mitigating virus-induced fetal growth retardation [171].

A different study has identified six ZIKV-specific nAbs that target key epitopes on the ZIKV E protein, including the C-C′ loop and the LR of the DIII domain (e.g., ZV-48, ZV-54, and ZV-67) [132]. These nAbs offer potential for targeted vaccine development by focusing on epitopes unique to ZIKV, thereby reducing the risk of cross-reactivity with DENV and minimizing the potential for ADE.

A second strategy involves modifying the Fc region of ZIKV-specific antibodies to prevent ADE while maintaining their neutralizing ability. In one study, researchers used a prior developed ZIKV-specific nAb ZV-54 targeting the LR of the DIII domain and then modified its Fc glycosylation profile to prevent interaction with Fcγ receptors on immune cells. The ZV-54 variants retained their neutralizing potency against ZIKV without triggering ADE in DENV infection, demonstrating a promising dual approach that preserves efficacy while eliminating ADE risk [172]. This approach showcases the potential of combining ZIKV-specific nAbs with targeted modifications to enhance vaccine safety.

Target-specific mutations in the ZIKV E protein may also improve the specificity of nAbs and reduce ADE risk. Several novel mutations in or near the FL of DII or DIII have been reported to decrease the production of cross-reactive antibodies [173]. These mutations may alter the antigenic structure of the E protein, making it more challenging for cross-reactive antibodies generated during DENV infection to enhance ZIKV infection. By improving the specificity of nAbs, these mutations can enhance the safety of vaccines by reducing the likelihood of ADE.

In addition to modifying the viral antigens, engineering nAbs to prevent their interaction with Fcγ receptors on immune cells offers another potential solution to ADE. This can be achieved through mutations in the Fc region of the nAbs, which block Fcγ receptor engagement and thereby prevent antibody-dependent viral entry into host cells [173]. Other techniques to limit ADE include blocking Fc receptor engagement, removing the antibody heavy chain, or deleting the N-linked sugar on IgG molecules, all of which prevent ADE while maintaining the neutralizing capacity of the antibodies [174].

In conclusion, strategies to reduce the risk of ADE in ZIKV vaccine development include identifying ZIKV-specific nAbs, modifying the Fc region of antibodies to avoid Fcγ receptor interactions, and introducing mutations in the ZIKV E protein to improve nAb specificity. These approaches are critical for creating safe and effective vaccines and therapies against ZIKV, while minimizing the potential for cross-reactivity with other orthoflaviviruses and reducing the risk of ADE.

## 6. Anti-ZIKV E Protein Inhibitors

As discussed in the previous section, vaccine development for ZIKV faces significant challenges, including cross-reactivity of nAbs, ADE, and antigenic diversity among different ZIKV lineages and other orthoflaviviruses [168]. Given these limitations, the discovery of inhibitors or host cellular restriction factors targeting the ZIKV E protein presents a promising alternative strategy to block viral entry and limit infection. As ZIKV E protein plays a critical role in mediating viral entry by facilitating multiple steps of virus–host interactions [1,86,88]. Depending on their mechanism of action, ZIKV E protein inhibitors can be categorized into four groups: (1) direct E protein inhibitors: molecules that interact with the E protein to block its function, (2) viral attachment inhibitors: agents that prevent ZIKV from binding to host cells by disrupting E protein interactions with cell surface receptors, (3) viral fusion inhibitors: compounds that block viral fusion with endosomes, thereby preventing genome release, and (4) host cellular restriction factors: cellular factors that interfere with ZIKV E protein-mediated viral entry. ZIKV E protein inhibitors can take various forms, including small molecules, natural compounds, proteins, and peptide-based inhibitors. Table 2 provides a comprehensive list of known inhibitors that target the ZIKV E protein or its associated viral entry processes. A brief summary of their mechanisms of action is outlined below.

### 6.1. Direct E Protein Inhibitors

Most of the direct E protein inhibitors reported so far are natural compounds with few exceptions. A small molecule F1065-0358 was found to inhibit ZIKV infection by binding to a region between DI and DIII of the ZIKV E protein, preventing its trimerization, a crucial step in the fusion process required for viral entry [175]. A natural compound, gossypol, a phenol derived from the cotton plant, exhibits potent antiviral activity against both African and Asian ZIKV strains. It neutralizes ZIKV infection by targeting DIII of the E protein. When combined with the anti-cancer drug bortezomib, it achieves a synergistic enhancement of antiviral activity [176,177]. Epigallocatechin gallate (EGCG), a natural compound found in green tea, binds to DI and DII of the ZIKV E protein, disrupting the conformational changes necessary for viral entry [178]. Pentagalloylglucose (PGG), a polyphenolic compound from the Chinese herb, inhibits ZIKV entry by interacting with charged residues on the viral envelope, such as glycosylated viral E proteins, thereby competing with natural virus–receptor interactions essential for viral binding [179]. Polysaccharides like Pentagalloylglucose, parishin, and stevioside inhibits the viral entry by binding the Zika virus envelope protein [180].

Other natural compounds include Apigenin, a flavonoid from the flavone glycoside class, which exhibits high affinity for the ZIKV E protein DIII and significantly reduces viral titers in Vero cells [181]. Similarly, baicalin, a glucuronide derivative of baicalein found in *Scutellaria baicalensis*, has demonstrated strong antiviral activity against ZIKV entry with high binding affinity to the E protein and low cytotoxicity [182]. Atranorin, a lichen-derived secondary metabolite, effectively blocks ZIKV entry and reduces infectivity in vitro by directly targeting the E protein, with docking studies suggesting optimal binding between DI and DIII. Additionally, it inhibits ZIKV infection in human glial cells by attenuating IFN signaling, further highlighting its potential as an antiviral agent [123]. Palmatine, a protoberberine alkaloid compound that inhibits ZIKV infection by interacting with the E protein, has been shown to destabilize ZIKV. It inhibited ZIKV binding and entry by 95% and 69%, respectively, likely through interaction with the ZIKV E protein, consistent with molecular docking analysis [183]. Lastly, harringtonine, a natural alkaloid from the Cephalotaxus genus, demonstrates nanomolar-level efficacy in inhibiting multiple stages of ZIKV infection, including stability, binding, entry, replication, and release, through direct interaction with the E protein [184].

### 6.2. Viral Attachment Inhibitors

Several attachment inhibitors, Cabozantinib, R428, TP-0903, and BMS-777607, significantly impaired ZIKV infection by inhibiting the AXL receptor in a human cerebral microvascular endothelial hCMEC/D3 cell line and the human umbilical vein endothelial cells (hUVECs) [185], which may serve as potential antiviral therapeutics for suppressing ZIKV infection [185,186]. Another small molecule attachment inhibitor ZINC33683341, identified through in silico modeling, has been shown to inhibit ZIKV entry by binding to primary receptors, with its antiviral activity confirmed in in vitro studies [187]. Curcumin, a component of turmeric, inhibits ZIKV attachment and entry by disrupting viral E protein function and blocking E-cell receptor interactions [188,189].

### 6.3. Viral Fusion Inhibitors

A number of inhibitors have been identified that target the viral fusion step of the ZIKV E protein during viral entry. Through a high-throughput screening method, known as ALPHAscreen, which is a competitive amplified luminescent proximity homogeneous assay, has been used to discover small molecule inhibitors of the ZIKV E protein. Seven lead compounds were identified to disrupt E protein-mediated membrane fusion during viral entry of ZIKV and DENV infections, thereby blocking the viral infection [190]. Moreover, the recombinant venom peptide rEv37, derived from the scorpion *Euscorpiops validus* and produced in a prokaryotic system, has shown to prevent the low pH-dependent fusion, thus restricting the late step of the viral entry into the host cell during ZIKV and DENV2 infection [191]. Atovaquone, a synthetic compound with antifungal antiparasitic properties [192,193] has been shown to suppress ZIKV and DENV1-4 infection in both mammalian and mosquito-derived cells [194]. Its antiviral activity is attributed to the inhibition of E protein-mediated membrane fusion during viral entry [194]. The synthetic peptide Z2 disrupts ZIKV virion integrity, inhibiting infection in vitro. Additionally, it crosses the placental barrier and prevents vertical transmission in C57BL/6 pregnant mice, possibly by inducing pore formation in the viral membrane through disruption of E protein conformational changes [195,196]. Another fusion inhibitor, P5, a peptide derived from the JEV E protein stem, effectively blocked ZIKV infection and conferred in vivo protection in AG6 mice by altering E protein conformation under low pH conditions [197].

### 6.4. Host Restriction Factors

Several host cellular factors restrict ZIKV infection, including LAMR1 (Laminin receptor 1), Hpa (heparanase), Viperin, and USP38 (Ubiquitin-specific peptidase 38), which target different stages of the viral life cycle, such as entry, replication, and protein stability. Specifically, LAMR1 suppresses ZIKV infection by interacting with the E protein and reducing its ubiquitination [198]. Hpa, a multifaceted protein and an endo-β-D-glucuronidase, degrades heparan sulfate and functions as a host restriction factor to attenuate ZIKV infection by destabilizing the viral E protein [199]. Viperin is an interferon-inducible protein, also known as virus-inhibitory protein, restricting the replication of a wide range of viruses including controlling the ZIKV infection [200]. Finally, USP38, a ubiquitin-specific peptidase, has been identified as a host restriction factor in resisting ZIKV infection by removing the ubiquitination of the viral E protein, which is critical for ZIKV infection and transmission [201].

**Table 2:** Anti-ZIKV E inhibitors.

Compound Name	Effect	IC_50_/EC_50_ (µM)	CC_50_ (µM)	Cell /Model	Reference
Direct E Protein Inhibitors					
F1065-0358	Bind to the DI and DIII regions and interfere with the E protein trimerization during	14	200	Vero	[175]
Gossypol	Bind to DIII	3.75 ± 0.01	14.17 ± 0.74	Vero E6	[177]
EGCG	Bind to DI and DII	na	na	Vero E6	[178]
PGG	Interacts with charged residues of glycosylated E protein	4.1	114	Vero B4	[179]
Polysaccharides (PGG, parishin and stevioside)	Bind to E protein	na	na	Docking analysis	[180]
Apigenin	Bind to DIII	>100	na	Vero	[181]
Baicalin	Bind to E protein	14	na	Vero	[181,182]
Atranorin	Bind to DI and DIII	11.9	>50	SNB-19	[123]
Palmatine	Interact with E protein	na	na	Vero	[183]
Harringtonine	Bind to E Protein	0.287	>10	Vero	[184]
**Viral Attachment Inhibitors**					
Cabozantinib (R428, TP-0903, and BMS-777607)	Inhibit AXL receptor	na	na	hCMEC/D3/HUVECs	[185]
ZINC33683341	Bind to primary receptors	na	na	Vero	[187]
Curcumin	Viral attachment and entry by abrogating the function of viral envelope proteins	1.9	11.6	Vero	[188,189]
**Viral Fusion Inhibitors**					
Seven compounds	Prevent E-mediated membrane fusion	0.9–19.3	5.2–>100	Vero	[190]
Ev37	Prevent viral membrane–endosomal membrane at low pH	na	116.3	Huh-7	[191]
Atovaquone	Block E-mediated membrane fusion	2.1	na	Vero/MDCK/C6/36	[194]
Peptide Z2	Disrupt E conformational changes	1.75	na	C57BL/6BHK21	[195,196]
P5	Change E protein conformation at low pH	3.27	na	Vero/AG6	[197]
**Host Restriction Factors**					
LAMR1	Attenuate E protein ubiquitination	na	na	HeLa/HEK293T	[198]
Hpa	Attenuates ZIKV infection by destabilizing the E protein	na	na	MEF	[199]
Viperin	Restrict a wide range of viruses including ZIKV	na	na	Huh7	[200]
USP38	Attenuates K48- and K63-linked polyubiquitination of E protein	na	na	HeLa/HEK293	[201]

Note: na, data not available.

## 7. Concluding Remarks

ZIKV infects host cells through an E protein-mediated viral entry mechanism that is a highly conserved process across humans, animals, and mosquitoes. However, the specific cell surface receptors required for viral attachment and fusion may vary among different hosts. These differences in receptor usage could contribute to variations in viral tropism and pathogenicity across species [97].

The GL, particularly the N154 glycosylation of the ZIKV envelope protein, plays a critical role in viral entry by facilitating attachment to host cell receptors and enhancing infectivity. Additionally, this glycosylation site contributes to immune evasion by modulating host immune recognition and response [105,202]. Therefore, the significant differences observed between African and Asian ZIKV lineages, particularly in glycosylation and protonation status, may influence cell tropism, viral entry, and pathogenesis in their respective hosts. The nAbs play a crucial role in neutralizing the ZIKV E protein activities during viral entry and the development of vaccines in mitigating ZIKV infection. Critical antigenic sites such as DI, DII, DIII, and combination of these domains are all major targets for nAbs. In particular, the most promising antigen site for nAb development is DIII, which not only induces potent nAbs but also causes minimal ADE [127,203,204]. Additionally, targeting the GL, the N154 glycosylation could also potentially be a promising strategy for nAb discovery because the GL is adjacent to the FL, potentially creating steric hindrance that inhibits virus entry and infection [1,143,205]. Note that although large numbers of nAbs and ZIKV E protein inhibitors and natural compounds have been reported, none of them have been approved by FDA to treat the ZIKV infection [206].

ZIKV remains a significant public health concern, particularly in regions with ongoing transmission and the potential for new outbreaks [15,207]. Given the virus’s ability to spread rapidly and the devastating consequences it can develop, such as neurologically related diseases in adults and congenital malformations such as microcephaly in newborn babies or during pregnancy, continuous surveillance and research are essential. ADE may also pose a challenge for ZIKV treatment and vaccine development because cross-reactive antibodies against DENV, which co-circulates with ZIKV, can enhance ZIKV infection instead of neutralizing it, potentially leading to more severe disease [167]. The emergence of new ZIKV variants could pose additional challenges for drug discovery and vaccine development, as mutations in viral proteins may affect transmissibility, pathogenicity, and immune escape [208,209]. Variants with alterations in the E protein could potentially evade nAbs generated from previous infections reducing the effectiveness of current therapeutic and preventive measures [71]. The risk of emerging variants is heightened by factors such as international travel, urbanization, and climate change, which may expand the habitats of *Aedes* mosquitoes, the primary vectors of ZIKV [210]. In this context, the need for robust public health strategies, including monitoring of viral evolution and preparedness for vaccine updates, is more critical than ever to mitigate the impact of future ZIKV outbreaks.

## Abbreviations

a.a.	Amino acids
ADE	Antibody-dependent enhancement
BBB	Blood-brain barriers
CDE	Clathrin-dependent endocytosis
CHIKV	Chikungunya virus
CIE	Clathrin-independent endocytosis
CPE	Cytopathic effect
CNS	Central nervous system
DC	Dendritic cell
DENV	Dengue virus
dsRNA	Double stranded RNA
EDE	E dimer epitope
EGCG	Epigallocatechin gallate
EM	Electron microscopy
ER	Endoplasmic reticulum
FL	Fusion loop
GBS	Guillain–Barré syndrome
GL	Glycan loop
HAVcr-1	Hepatitis A virus cellular receptor 1
hBMEC	Human brain microvascular endothelial cell
hEC	Human epithelial cell
hNPC	Human neural progenitor cell
hUVEC	Human umbilical vein endothelial cells
IFN	Interferon
IRF3	Interferon regulatory factor 3
ISGS	Interferon stimulated genes
JEV	Japanese Encephalitis virus
LAMR1	Laminin receptor 1
LR	Lateral ridge
mAb	Monoclonal antibody
MEF	Mouse Embryonic Fibroblasts
NAb	Neutralizing antibody
NPCs	Neural progenitor stem cells
NSCs	Neural Stem Cells
PAMP	Pathogen-associated molecular pattern
PGG	Pentagalloylglucose
PRRs	Pattern recognition receptors
PtdSer	Phosphatidylserine
RdRP	RNA-dependent RNA polymerase
RIG-1	RIG-1-like receptor
RLRs	RIG-I like receptors
ROS	Reactive oxygen species
sfRNA	Subgenomic flavivirus RNA
TAM	Tyro3, Axl and Mer
TGN	Trans-Golgi network
TM	Trans-membrane
TLR	Toll-like receptors
TOR	Target of rapamycin
WHO	World health organization
WNV	West Nile virus
YFV	Yellow Fever Virus
ZIKV	Zika virus
